# Supplementary material for: CardamomOT: A mechanistic optimal transport-based framework for gene regulatory network inference, trajectory reconstruction and generative modeling
Source: PLoS Comput Biol. 2026 Oct 6;22(10):e1014838. doi: 10.1371/journal.pcbi.1014838 (PMC13686291; doi:10.1371/journal.pcbi.1014838)
Supplement: Supplementary file 1 [file pcbi.1014838.s016.pdf]

Response to Reviewers

CardamomOT: a mechanistic optimal transport-based framework for gene regulatory network inference, trajectory reconstruction and generative modeling

Manuscript Number: PCOMPBIOL-D-26-00826

Yann Maugé and Elias Ventre

August 14, 2026

We thank the Editor, Section Editor, and all four Reviewers for their careful and constructive reading of our manuscript. Their comments have helped us identify important improvements in clarity, validation, and presentation. Below we address each point individually. Changes to the manuscript are highlighted in the revised version (*Revised Manuscript with Track Changes*).

Response to the Editor — Journal Requirements

Ed.1 | Journal Requirement — Manuscript source file

Please upload your manuscript file as a .doc, .docx, .rtf or .tex (LaTeX Source File).

Response to Ed.1

We have uploaded the manuscript source as a `.tex` file under the item type "LaTeX Source File", with the corresponding PDF as "Manuscript".

Ed.2 | Journal Requirement — Figure files

Please upload all main figures as separate Figure files in .tif or .eps format.

Response to Ed.2

All main figures have been exported as separate `.eps` files and added to the submission.

Ed.3 | Journal Requirement — Supporting Information

Supplementary Figures and Tables are included in the manuscript file. Please remove them and upload them with the file type "Supporting Information", with a legend listed in the manuscript.

Response to Ed.3

Supplementary figures and tables have been removed from the main manuscript file and uploaded as separate Supporting Information files. Legends for each Supporting Information item have been added to the manuscript after the reference list.

Ed.4 | Journal Requirement — Financial Disclosure

Please amend your detailed Financial Disclosure statement (full sentences, initials alongside each funding source, role of funders).

Response to Ed.4

The financial disclosure statement has been amended as follows: "This work was supported by an ENS de Lyon CDSN PhD fellowship awarded to YM. The funders had no role in study design, data collection and analysis, decision to publish, or preparation of the manuscript."

Additional changes

We detail here a modification that has been done during the revision process, independently on what the reviewers had noticed:

Extension of CardamomOT to multiple explicit stimulus nodes. The Schiebinger reprogramming dataset involves two biochemically distinct perturbation phases: doxycycline-induced OSKM expression (days 0–8) and serum/LIF culture conditions (days 0–18). In the previous version of the algorithm, CardamomOT was limited to a single constant stimulus node, which conflated these successive perturbations into an averaged effect whose inferred targets lacked clear biological interpretability. We have now extended the algorithm to support multiple simultaneous stimulus nodes, each with a user-defined activity window, and re-analyzed this dataset accordingly. Two stimulus nodes were introduced—*Dox* and *Serum*—yielding biologically distinct and more interpretable regulatory programs for each perturbation (Figure S3, E,G). The *Dox* node captures the absence of activation of late pluripotency markers such as *Dppa5a* and the epithelial gene *Cldn7*, consistent with the early reprogramming phase preceding the mesenchymal-to-epithelial transition. The *Serum* node drives repression of *Lox*, a pro-EMT enzyme whose suppression is known to facilitate mesenchymal-to-epithelial transition during reprogramming, as well as genes associated with inflammatory signaling (*Ereg*, *Tnfrsf12a*). This decomposition demonstrates that CardamomOT can now disentangle the contributions of successive or co-occurring perturbations, improving both the biological interpretability of inferred GRNs and the internal consistency of the model. This re-analysis affects all Schiebinger-derived results (Figures 2, 6, 7, S3–S5, S10 and Table S3), and we have verified that it leaves every conclusion of the original submission unchanged — in particular the predicted enhancement of iPSC-like conversion under Obox6 and Zfp42 overexpression, with an unchanged magnitude of effect. Obox6 additionally now ranks among the top candidate hub regulators (Table S3). The manuscript and supplementary figures have been updated accordingly.

Response to Reviewer #1

We thank Reviewer 1 for a thorough and rigorous evaluation of the manuscript. We address each major and minor comment in turn.

Major Comments

R1.1 | Benchmark fairness should be clarified

The synthetic data appear to be generated from a model closely related to the assumptions of CardamomOT, which may favour the proposed method. The authors should either add benchmarks with model misspecification or explicitly frame the synthetic benchmarking as validation under model-matched conditions.

Response to R1.1

This is a fair and important point. The simulated benchmarks are generated from the same mechanistic model underlying CardamomOT (via the HARISSA package), constituting a *model-recovery test* rather than a fully general benchmark. We have clarified this framing explicitly in Section 2.1: the simulated benchmarks establish best-case performance when model assumptions are satisfied, and all competing methods are evaluated under identical conditions.

Benchmarks under model misspecification are an important direction for future work. We partly addressed this by testing the robustness of CardamomOT to technical noise and irregular temporal sampling in our new analysis *(see also our responses to: R2.6, R4.5)*

R1.2 | Uncertainty in inferred GRNs should be reported

Experimental GRNs are presented as point estimates. The authors should report edge stability or confidence, for example using bootstrapping, subsampling, or repeated random initializations.

Response to R1.2

We agree that edge uncertainty is important for biological interpretation. We have added:

- A supplementary figure (Figure S9) showing GRN stability across repeated runs with different random seeds for the experimental datasets.
- Expanded Discussion (Scalability and identifiability) on run-to-run variability as a diagnostic for identifiability and guidance for use.

R1.3 | Perturbation predictions should be more cautiously interpreted

The distinction between predictions supported by independent experimental evidence and purely model-generated hypotheses should be made clearer. Effect sizes and variability across repeated simulations would be more informative than only reporting chi-squared tests.

Response to R1.3

We have revised Section 2.6 to improve distinction between predictions consistent with prior experimental findings or molecular functions (Dnmt3a OV in the Semrau dataset; Obox6 and Zfp42 OV in the Schiebinger dataset), and model-generated hypotheses awaiting experimental validation (CHGA/STMN2 perturbations in the Kameneva dataset).

Figure 7 now includes error bars indicating variability across 5 independent repeated simulations per perturbation alongside the χ^2 test results, which provide a more interpretable measure of biological relevance than p-values alone.

R1.4 | Claims moderation

Several claims should distinguish validation on simulated data from consistency checks on experimental data. Statements such as "accurately recovers velocity fields", "accurately infers GRNs", and "reliable GRN structures" should be moderated when referring to experimental datasets.

Response to R1.4

We have revised the Abstract, Author summary, and Results sections throughout to consistently distinguish:

- *Simulated data*: quantitative claims are retained where ground truth is available (e.g. “cosine similarity > 0.8 on benchmark networks”).
- *Experimental data*: replaced with consistency-based language (e.g. “the calibrated model reproduces the observed expression dynamics”, “the inferred GRN is consistent with known regulatory roles”).

The Abstract now reads: “We validate our framework on both in silico and experimental datasets, demonstrating computational scalability and consistently improved performance over state-of-the-art methods in both GRN and trajectory reconstruction on simulated datasets, and, on experimental datasets, reconstruction of cellular trajectories, velocity fields and latent protein levels that are mutually consistent, together with GRN structures consistent with known biology.”

Minor Comments

R1.5 | Inconsistent terminology: “gene regulation network” vs “gene regulatory network”

The authors use both terms. Please choose one.

Response to R1.5

We have standardised the terminology throughout to “gene regulatory network” (GRN). The keyword list has been updated accordingly.

R1.6 | Hybrid stochastic-deterministic notation in Eq. (2)

The two-state model is described as stochastic for both $M_i(t)$ and $P_i(t)$, but Eq. (2) combines stochastic dynamics for M_i production with mean-field ODEs for degradation and P_i. Please revise or explain this hybrid notation.

Response to R1.6

The model in Eq. (2) is indeed a *hybrid stochastic-deterministic* (piecewise deterministic) model: mRNA production occurs via a Poisson process driven by burst events (stochastic), while degradation of M_i and the dynamics of P_i are deterministic ODEs. This is the standard formulation of the two-state telegraph model in the bursty regime, consistent with the framework of Piecewise Deterministic Markov Processes (PDMPs).

We have added a clarifying sentence in Section 1.1 making the hybrid nature of Eq. (2) explicit, and added a notation remark distinguishing stochastic jump terms (written with $\to$) from deterministic flow terms (ODEs).

R1.7 | Define notation $\varepsilon(k_{\text{off}}/s_0)$

E.g. does this denote an exponential distribution?

Response to R1.7

Yes — $\mathcal{E}(k_{\text{off}}/s_0)$ denotes an *exponential distribution* with rate parameter k_{off}/s_0 (mean burst size s_0/k_{off}). We have added an explicit definition at first use in Section 1.1.

R1.8 | Define the negative binomial parameterisation in Eq. (3)

Different conventions use different meanings for the second parameter.

Response to R1.8

We agree, and we have added an explicit definition at first use in Section 1.1. We write NB(a, b) for the Gamma–Poisson mixture defined by $\lambda \sim$ Gamma(shape a, rate b) and $M \mid \lambda \sim \mathcal{P}(\lambda)$, so that in Eq. (3)

$$a = \frac{k^{\theta}_{\text{on},i}(P(t))}{d_{0,i}} \quad \text{is the } \textit{shape} \text{ parameter (burst frequency relative to the mRNA degradation rate),}$$

$$b = \frac{k_{\text{off},i}}{s_{0,i}} \quad \text{is the } \textit{rate} \text{ parameter (inverse mean burst size).}$$

This convention gives $\mathbb{E}[M_i] = a/b = \left(k^{\theta}_{\text{on},i}(P(t))/d_{0,i}\right)\left(s_{0,i}/k_{\text{off},i}\right)$ and a Fano factor $1 + s_{0,i}/k_{\text{off},i}$, i.e. the classical result that mean expression is the product of burst frequency and burst size, while overdispersion is controlled by burst size alone. For readers using the size–probability convention, this is equivalent to a negative binomial with size a and probability $b/(1 + b)$. We have added this equivalence explicitly in the text so that the parameterisation is unambiguous under either convention.

R1.9 | Additional peer-reviewed source for the NB approximation

Could the authors provide an additional peer-reviewed source, in addition to the PhD thesis currently cited [31]?

Response to R1.9

We have added peer-reviewed references supporting the NB approximation, including Zong et al. (2010, *Mol. Syst. Biol.*) and Grün et al. (2014, *Nature Methods*), cited alongside [31] at first use.

R1.10 | Algorithm 1: should there be $N - 1$ OT problems?

Apologies if my indexing is wrong — should there be $N - 1$ OT problems rather than N?

Response to R1.10

The reviewer is correct. For N timepoints, one OT problem is solved per consecutive pair (t_j, t_{j+1}), yielding $N - 1$ problems. Algorithm 1 has been corrected.

R1.11 | Abbreviations: define at first use, use consistently

E.g. “Reference Fitting (RF). Reference Fitting” appears redundant.

Response to R1.11

We have reviewed all abbreviations and ensured they are defined at first use and used consistently thereafter. The redundant occurrence has been corrected.

R1.12 | State number of simulations and meaning of error bars in captions

Please state number of independent simulations and what error bars/bounds represent in figure captions.

Response to R1.12

We modified figure captions to include the number of independent simulations and the nature of error bars (standard error of the mean or standard deviation, as appropriate) consistently.

R1.13 | Figure 7: detail on reconstructed cell counts for χ^2 tests

E.g. are all $p < 10^{-3}$ because you ran 1000 simulations?

Response to R1.13

We thank the reviewer for this remark. We agree that p-values computed from the raw simulated cell counts would be strongly influenced by sample size. We have therefore changed the way the χ^2 statistic is computed in the current version to decouple it from the number of simulated cells. The test compares the cell-type distributions of Sim perturb and Sim single. Rather than using the raw simulated cell counts, which range from a few thousand cells for Semrau and Kameneva to ∼250,000 for Schiebinger, we rescale the compared proportions to a fixed pseudo-count of 100 cells per cell type before constructing the contingency table. This provides a common scale for comparing the magnitude of compositional shifts across datasets, independently of their original sample sizes. We have made explicit in both Section 2.6 and the caption of Figure 7 that this rescaled statistic is used as a comparative measure of compositional separation rather than as a formal inferential test on the underlying biological system, since the pseudo-count is fixed by convention rather than derived from the data. Effect variability is reported separately as error bars over $N = 5$ independent stochastic replicates. Under this revised analysis, CHGA KO alone no longer produces a shift clearly distinguishable from the run-to-run variability of the simulations, and we have adjusted the corresponding statement in Section 2.6 accordingly.

R1.14 | "Digital twin" terminology may not be appropriate

The model does not appear to involve iterative updating between the model and the biological system.

Response to R1.14

We agree. "Digital twin" implies bidirectional feedback between a model and a physical system, which is not the case here. We have replaced it with " the calibrated model functions as an in-silico representation of the biological system", which more accurately describes the CardamomOT output.

R1.15 | Dataset accession numbers missing from Data and Code Availability

Please ensure that all experimental datasets have clear accession numbers, not only a GitHub link.

Response to R1.15

Explicit accession numbers have been added to the Data and Code Availability section:

- Semrau et al. (2017): GEO accession GSE79578.
- Kameneva et al. (2021): GEO accession GSE147821.
- Schiebinger et al. (2019): GEO accession GSE106340 and `https://singlecell.broadinstitute.org/single_cell/study/SCP295/`

These have also been added to the GitHub README.

Response to Reviewer #2

We thank Reviewer 2 for their careful attention to presentation and methodological assumptions.

R2.1 | Figure 1: mixed vector/raster rendering

Figure 1 could be cleaned up: it appears to be a jarring mix of vector text and raster equations of varying resolution; panel B seems to have vector axes but low-resolution raster points.

Response to R2.1

We have regenerated Figure 1 so that all elements (text, equations, data points) are rendered as vectors. The figure has been re-exported and uploaded.

R2.2 | Figure 1 panels B and E: unclear relationship

I am unsure what panels B and E represent in relation to each other. Panel E (predictive trajectories) seems to be a subset of the data points of panel B (experimental plot), when I would expect them to be switched.

Response to R2.2

Panels B and E contrast the *input* (B: raw experimental snapshots) with the model *output* (E: simulated trajectories from the calibrated model). We have revised the figure layout to make this contrast visually explicit, with

- Clear “CardamomOT input” / “CardamomOT output” labels.
- Redrawing the ”cell” points to explicit resimulation at step E and avoid confusion due to the previous similarity of the cells.

R2.3 | Figure 3: “Trees” box border clipped

In Figure 3, the “Trees” box border has been clipped.

Response to R2.3

This has been corrected in the revised Figure 3.

R2.4 | Validity of piecewise-linear interpolation outside the adiabatic regime

A considerable emphasis is placed on the model's extension to a non-linear regime. Is there a flaw in the methodology from discretising the trajectories into linear segments using the piecewise constant parameter α (Appendix A)? The justification seems to rest on the asymptotic regime where the protein timescale $\ll$ mRNA degradation, but what about outside this regime?

Response to R2.4

The reviewer's question rightly pointed to a step that deserved to be stated explicitly rather than left implicit, and we have restated it more carefully than in the original submission. Eq. (15) in Appendix A is not a numerical discretization of a nonlinear ODE. Within an interval, we replace the protein-dependent burst frequency $k^{\theta}_{\mathrm{on},i}(P(t))$ by a piecewise-constant driving term taking the two endpoint basin modes, with a single switch; under that substitution the protein ODE (5) is linear and Eq. (15) is its exact closed-form solution, so no additional discretization error arises from the formula itself. We should however be explicit that the substitution *is* the approximation, and it has two parts: the regulatory feedback is frozen within the interval, and a single switch per gene is assumed to describe the interval.

What we would emphasize is the scope of this piecewise-constant treatment, which we had not made clear enough. It applies only to the construction of candidate protein paths between two observed timepoints. The nonlinear GRN-driven structure is retained wherever the network is estimated or used: in the OT cost of Eq. (8), in the GRN regression of Eq. (10) and the basin refinement of Eq. (11), in the kinetic recalibration of Section 1.2.3, where the full nonlinear field is integrated with an adaptive-step solver and no piecewise-constant substitution, and in the generative simulations, which sample the complete PDMP (2). The linearity of Eq. (15) is a local device for generating transport candidates, not a linearization of the inferred dynamics; the contrast with a linear reference process such as RF's Ornstein–Uhlenbeck model therefore stands.

Both parts of the approximation are justified by the metastable, bursty and adiabatic regime already required for the mechanistic model of Section 1.1 (see also our responses to: R4.8, R4.3): within a basin the driving mode is close to constant, and transitions between well-separated basins are rare and fast. Outside this regime, the discrete-basin representation underlying the entire method — the Negative Binomial mixture and its basin labels, not just the protein interpolation — ceases to describe the data before the interpolation itself becomes the binding issue: the two approximations share a regime of validity and fail together, not independently. We have rewritten the *Regime of validity* paragraph of Appendix A accordingly.

R2.5 | n_{pas}: choice in practice and effect of coarse sampling

The linear-spaced sample set (line 904) raises questions: (a) how is n_{pas} determined in practice? (b) What effect does a coarse sample space have on the misidentification of candidate cells and therefore the trajectory?

Response to R2.5

(a) n_{pas} is the number of grid points used to search for the switching time $\alpha_{c,i}$ within an inter-timepoint interval (Appendix A). The default is $n_{\mathrm{pas}} = 25$, and the implementation automatically increases it so that at least one grid point falls within each time unit of

$\Delta t = t_{j+1} - t_j$; this default was used, unmodified, for every benchmark and experimental dataset in this article, so in practice this is not a parameter users need to tune.
(b) The effect of a coarser grid is bounded and, in the regimes relevant to this work, negligible. Given a fixed switching gene, Eq. (15) varies smoothly in α, so a coarser grid only introduces a smooth interpolation error in the reconstructed protein value rather than a discontinuous misassignment. More importantly, the search only needs enough resolution to determine on which side of the mode-crossing time the interval falls (Appendix A, item 3). Finally, $\alpha_{c,i}$ is not estimated once: it is refined at every iteration of the EM loop jointly with the GRN, so any residual error from a coarse initial grid tends to be corrected over subsequent iterations rather than propagating to the final trajectories. We have added a remark to this effect in Appendix A. We would expect this parameter to become relevant only in a pathologically coarse regime (n_{pas} of a few grid points, effectively removing timing resolution within the interval), which is well below both the default and the automatically enforced minimum.
To illustrate this, we performed a sensitivity analysis under n_{pas} refinement as proposed by the reviewer (Figure S11). Performance plateaus from $n_{\text{pas}} \approx 4$ onwards; only the extreme value $n_{\text{pas}} = 2$, which removes essentially all timing resolution within an interval, degrades accuracy, consistent with the pathologically coarse regime anticipated above. The default of 25 thus sits far inside the plateau.

R2.6 | Potential bias from timestep choice in benchmarks

The choice of timestep influences the switching parameter sample set. (a) More detail on the choices of times and n_{pas} used in benchmark generation. (b) Sensitivity analysis of Figure 3 results under timestep and n_{pas} refinement.

Response to R2.6

(a) The benchmark timepoints (0–96 h) and $n_{\text{pas}} = 25$ were fixed prior to any comparisons and are identical for all methods. Competing methods do not use n_{pas} at all, so there is no differential tuning. These choices are fully documented in the code repository.

(b) We have added a sensitivity analysis (Figure 4H) showing GRN inference AUPR under increasing variability in the inter-timepoint intervals: for each replicate, the gaps between consecutive timepoints are drawn from a Dirichlet distribution with the endpoints of the time course held fixed, so that $\eta = 0$ recovers equispaced snapshots and $\eta = 1$ corresponds to uniformly sampled ones. Randomising the gaps at fixed endpoints necessarily produces replicates in which some consecutive timepoints are widely separated — precisely the configurations in which the switching-parameter sample set has to resolve a transition spread over a long interval. It also mimics the situation faced experimentally, where the number of snapshots is fixed by budget and the times at which the informative transitions occur are not known in advance, so that some intervals inevitably straddle them. AUPR is stable across the range of η, with slightly increased variability at high η indicating that the benchmark results do not depend much on a favourable placement of the sampling times.

We should be explicit about what this analysis does not vary: the total number of snapshots is held constant, so it probes the placement of the timepoints rather than a uniform refinement or coarsening of the time course as a whole. We would expect the latter to be governed by the temporal-resolution limitation already discussed in Section 3 — namely that intervals containing several mode switches per gene fall outside the single-switch assumption of Appendix A — rather than by any interaction with n_{pas},

since n_{pas} is automatically increased with Δt so that grid resolution is preserved as intervals lengthen.

R2.7 | Nuanced discussion of the method hierarchy in Figure 3

Should the reader conclude that CardamomOT is currently the best method and CARDAMOM the second best? It is difficult to discern what is responsible for this hierarchy — optimal transport minimising squared-Euclidean cost appears worse than neglecting OT mechanics altogether.

Response to R2.7

We agree this ordering is easy to misread, and we have added a dedicated paragraph to Section 2.3 to make the explanation explicit. The hierarchy in Figure 3A should not be read as a ranking of methods in general, and in particular not as evidence that OT-based trajectory inference is counterproductive; it reflects how well each method's dynamical and statistical assumptions match the process that generated the benchmark data.
CARDAMOM never reconstructs trajectories, but its per-timepoint statistical model is the Negative Binomial approximation derived from the very mechanistic model used to simulate the benchmarks (Section 2.1), so it is, in this specific sense, correctly specified for these data and can recover accurate parameter estimates without tracking cells across timepoints. RF does reconstruct trajectories, but under a linear Ornstein–Uhlenbeck reference process that neither exploits this NB structure nor captures the nonlinear, GRN-driven dynamics of the benchmark networks; this mismatch degrades both the inferred trajectories and the GRN subsequently built from them. CardamomOT is designed precisely to remove this trade-off, by pairing explicit trajectory reconstruction with the same nonlinear mechanistic reference process instead of a misspecified linear one — which is why it outperforms both. The general statement is therefore that the value of an OT step depends on how well the reference process matches the true dynamics, not on the presence or absence of trajectory inference per se.
We emphasize that the `noloop` ablation of Figure 4A–E, which performs a single round of OT on the burst modes without the iterative refinement loop, supports this reading directly: its accuracy is lower than that of the full method, substantially so on BN8, yet it remains above RF on the same networks (compare Figure 4 and Figure 3A).

Response to Reviewer #3

We thank Reviewer 3 for their positive assessment and constructive suggestions.

R3.1 | General assessment and recommendations

The manuscript is well organised and the methods are described in sufficient detail to allow reproduction. The results on simulated benchmarks are convincing. The application to experimental datasets is promising, although the support there is somewhat more qualitative. It would strengthen the manuscript to include more quantitative evaluation on experimental data where feasible, and a brief discussion of sensitivity to key hyperparameters and model assumptions. Overall, a strong contribution; recommend acceptance after minor revision.

Response to R3.1

We thank Reviewer 3 for this positive evaluation. In response to the suggestions:

- More quantitative evaluation on experimental data: we have added stability analysis for experimental GRNs *(see also our responses to: R1.2)*, perturbation effect sizes with variability across runs *(see also our responses to: R1.3)*, and PHATE embeddings as an additional dimensionality reduction method *(see also our responses to: R4.1)*.
- Hyperparameter sensitivity and model assumptions: We discuss sensitivity to protein degradation rates in more details *(see also our responses to: R4.3)*, as well as robustness towards data modulations *(see also our responses to: R2.6, R4.5)* and provide guidance for users in the discussion.

Response to Reviewer #4

We thank Reviewer 4 for a detailed and constructive review. Several major suggestions have led to meaningful improvements in the manuscript.

Major Comments

R4.1 | Robustness of low-dimensional embedding validation

Several validation analyses rely on visual comparison in UMAP space. The authors should repeat key visualizations using an additional trajectory-preserving method such as PHATE.

Response to R4.1

We have added PHATE embeddings as supplementary figures (Figure S10) for all three experimental datasets, showing both reference and simulated data. The PHATE embeddings confirm the conclusions drawn from UMAP: the CardamomOT simulated data preserves the global trajectory structure and temporal ordering of the reference data. PHATE embeddings were computed from the concatenated reference and simulated datasets, with each plotted separately, consistent with our UMAP methodology.

R4.2 | Gene subset selection and possible selection bias

The manuscript would benefit from a clearer discussion of how gene subsetting may affect the inferred networks, and whether CardamomOT can be run on substantially larger gene sets.

Response to R4.2

We have added explanations regarding these points in Section 3:

- **Gene selection introduces biases, and is itself prior information.** We have added a dedicated paragraph to the Discussion (“Gene selection and its influence on inferred networks”). It first discusses how the choice of gene set shapes the inferred network: the influence of an omitted regulator is redistributed among the retained genes, so that inferred edges may summarise indirect regulation through unobserved intermediates; and selecting genes from known markers or previously characterised modules, as for the Semrau and Kameneva datasets, biases the network toward well-characterised regulatory axes. We now state explicitly that the agreement between

our inferred hub regulators and those reported in the original studies is therefore a consistency check on a pre-selected gene set rather than an unbiased recovery of the underlying network, and we note that purely statistical selection criteria are not exempt either.
We then argue that the gene set should be recognised as a form of prior information in its own right, on the same footing as prior constraints on individual edges, and give a practical recommendation: use prior biological knowledge of the system, or the hypotheses motivating the analysis, to designate a core set of nodes of interest, then assemble the regulatory context of those nodes semi-automatically by combining curated interaction databases with data-driven discovery — for instance a genome-wide scalable method, used to obtain a coarse-grained approximation of the global network from which the neighbourhood of the core set is retained. Automating this construction is the object of ongoing work in our team.

- **Scalability, and the trade-off with prior structure.** Table S2 reports runtimes up to 100 genes; as discussed in the Discussion ("Scalability and identifiability"), the binding constraint beyond this scale is identifiability rather than computation, since the number of GRN parameters grows as n^2. We now make explicit in the new paragraph that this ceiling takes the practical form of a trade-off between gene-set size and the amount of prior structure imposed on the edges: in the fully unconstrained setting we recommend not exceeding the order of 100 genes, whereas accepting more prior structure — through a prior network θ^0 or zeroed entries — allows a correspondingly larger gene set for a comparable level of reliability.

R4.3 | Accuracy and availability of kinetic parameters in realistic applications

What level of uncertainty is typical for protein degradation-rate estimates in the systems considered? Do literature-derived estimates generally fall within the uncertainty range where the method remains robust?

Response to R4.3

We have modified the relevant paragraph in Section 2.3 connecting Figure 4F to realistic biological settings. Protein half-lives in mammalian cells typically range 40–100h with a larger range up to 10–200 h for more extreme cases (Dear et al. 2024, *PNAS*; Schwanhäusser et al. 2011, *Nature*). As an example of uncertainty, Rolfs et al. (2021, *Nature Communications*) report ±0.9d of median uncertainty in half-life in their mouse tissue atlas of 3106 proteins. Figure 4F shows CardamomOT maintains high AUPR up to ±50% perturbation. Typical uncertainties correspond to roughly ±50 % relative error on the half-life for the most common range, i.e. at the boundary of the regime where accuracy is preserved; we therefore also report robustness up to ±400 %, and note that performance remains well above baseline throughout.

R4.4 | Additional perturbation predictions for less-characterized genes

To demonstrate discovery potential, could the authors provide a proof-of-concept showing that CardamomOT can identify less-characterized genes with potentially important regulatory roles?

Response to R4.4

We have added a supplementary table (Table S3) listing the top candidate hub regulators by inferred outgoing regulatory strength for each experimental dataset, and updated the text in Section 2.3 and Section 2.6 accordingly.

We agree that the perturbations tested in silico in the original submission — *Dnmt3a* and *CHGA*, identified as high-confidence candidate regulators before any perturbation experiment, and *Obox6* and *Zfp42*, whose predicted effect on reprogramming efficiency matches published experimental validation [15] — do not by themselves constitute a proof-of-concept for *less-characterized* genes, since they were selected precisely because independent validation was already available. To address this directly, Table S3 also surfaces regulators with little or no established role in the corresponding differentiation process, which we now discuss explicitly in Section 2.6: for example *HAND2-AS1*, a long non-coding RNA antisense to *HAND2*, in the Kameneva sympathoadrenal network, and *Dmrtc2* and the poorly annotated *Gm26917* in the Schiebinger reprogramming network. These candidates were not selected on the basis of prior functional knowledge, and their co-occurrence with experimentally validated regulators in the same ranked output illustrates the potential of CardamomOT to generate testable hypotheses about previously uncharacterized genes, in addition to recovering known regulatory relationships. As in the original submission, we frame all candidates listed in Table S3 as testable hypotheses, and note explicitly that experimental validation of the newly proposed candidates is beyond the scope of this work.

We also note, as an illustration of the care required when interpreting such ranked outputs, that a subset of top-ranked candidates in the Kameneva dataset (e.g. *RPL30*, *ATP5F1E*) correspond to highly and uniformly expressed housekeeping genes rather than plausible regulators; we flag this explicitly in Section 2.3 so that ranked outputs of this kind are read alongside biological plausibility rather than taken at face value.

R4.5 | Clarification of simulated-data noise model

It is not fully clear whether simulations include technical noise resembling scRNA-seq measurement noise. Could the authors clarify and, if not, add a robustness analysis?

Response to R4.5

The simulated data generated by HARISSA include *intrinsic* biological stochasticity (from the two-state model) but not *technical* noise. We have added a supplementary robustness analysis (Figure 4G) introducing 7 increasing levels of binomial dropout imitating scRNA-seq noise. CardamomOT maintains high AUPR scores with slightly increasing variability at high levels of dropout, confirming robustness to moderate technical noise. We updated text accordingly in Section 2.3 and Section 2.1. *(see also our responses to: R1.1.)*

R4.6 | Usability and documentation of the software package

The documentation is currently difficult to navigate. The authors should consider hosting structured online documentation (e.g. ReadTheDocs) with clear separation of installation, tutorials, API documentation, and worked examples.

Response to R4.6

We have restructured the CardamomOT GitHub repository and created a ReadTheDocs documentation site at `https://cardamomot.readthedocs.io` (link added to the Data and Code Availability section). The documentation now includes:

- Installation guide
- Quick-start tutorial reproducing the main results of the paper
- API reference (auto-generated from docstrings)
- Three worked examples corresponding to the three experimental datasets

Minor Comments

R4.7 | Degradation missing from line 168 description

Around line 168, mRNA and protein degradation should also be mentioned alongside activation, synthesis, and production.

Response to R4.7

Revised to: "[. . .] the stochastic interplay between gene activation, mRNA synthesis, protein production, and their respective degradation processes."

R4.8 | Bursty regime assumption: does it hold for all genes?

Around line 180, is the bursty regime ($k_{\text{on}} \ll k_{\text{off}}$) expected to hold for all genes? What fraction can reasonably violate it?

Response to R4.8

The bursty regime is well supported for the majority of mammalian genes (Suter et al., 2011; Rodriguez & Larson, 2020, already cited). Constitutively expressed genes may not satisfy $k_{\text{on}} \ll k_{\text{off}}$; for these, the NB approximation remains valid but the two-mode approximation is less appropriate. We have amended Section 1.1 to note this limitation and point to the softmax extension (multi-mode model) as a potential solution.

R4.9 | NeuralODE recalibration step (line 302) is difficult to follow

It would be easier to follow if more details on the Neural ODE part could be provided.

Response to R4.9

We thank the reviewer for pointing out this gap. We have substantially expanded Section 1.2.3 to clarify that this step, previously described as NeuralODE recalibration and renamed "kinetic rate recalibration via differentiable ODE" for the sake of clarity, is not a generic black-box network but a direct parametrization of the mechanistic protein field of Eq. (5), with the converged GRN θ^* frozen and only the protein degradation rates d_1 (plus a small per-gene scale correction) treated as trainable. We now explicitly describe: (i) the model form, integrated with a differentiable adaptive-step ODE solver (`torchdiffeq`); (ii) the teacher-forcing training procedure, in which each consecutive timepoint pair is integrated starting from the *observed* reconstructed protein state rather than from the model's own earlier prediction, preventing compounding integration error, following standard practice

for single-cell dynamical models (PRESCIENT [45], STORIES [46]); (iii) the explicit loss function, combining reconstruction MSE with shrinkage of d_1 toward its literature prior; and (iv) the companion recalibration of the mRNA degradation rate d_0, which we now show is a closed-form, regularized method-of-moments estimator of $\tau = d_1/d_0$ derived from Campbell's theorem for the underlying bursty PDMP, rather than an unspecified variance-fitting step. A pseudocode block (Algorithm 2) summarizing the procedure has been added.

R4.10 | Reference needed for hub-regulator claim (line 460)

The statement that sparse networks with hub regulators are a hallmark of biologically realistic network structure should be supported by a reference.

Response to R4.10

We have added references supporting this observation (Ouma et al. 2018, *Plos Computational Biology* and Sanguinetti et al. 2019, book chapter).

R4.11 | Long section on experimental GRN inference without a main-text figure

The section beginning around line 453 is long and contains several important results on experimental GRN inference without a main-text figure. Consider shortening or moving a supplementary figure into the main text.

Response to R4.11

We agree, and have restructured this material rather than moving one of the existing supplementary figures into the main text. The reason is that the length of this section came mainly from descriptive passages — the gene selection criteria, the definition of the virtual stimulus nodes, and a detailed walkthrough of their inferred targets — rather than from results that a figure would help the reader interpret. Promoting Figure S1–S3 to the main text would have added a large multi-panel figure per dataset without shortening the text, and the quantitative results now most relevant to this section (reproducibility across runs, ranked hub regulators) are already reported in Figure S9 and Table S3, both added in response to Reviewer 1.

We have therefore moved the descriptive material to a new Appendix C ("Details on GRN inference for experimental datasets"), which collects the gene selection procedure, the definition and role of the stimulus nodes in each dataset, and the consistency check between their inferred targets and the known biology of each protocol. The main text now retains only the results themselves: network sparsity and hub structure, reproducibility across independent runs, the identity of the top regulators and their agreement with the original studies, and the non-negligible diagonal coefficients. This substantially reduces the section while keeping every result, and each condensed statement points to the appendix for the corresponding detail.

We also note that the inferred experimental GRNs are already represented in the main text, in the left panels of Figure 7, which show the regulatory neighbourhood of each perturbed gene in the corresponding dataset; Section 2.3 is thus not without a main-text visual counterpart, and the section it feeds into is figure-supported.

R4.12 | Figure 6 panels J–O: legend missing

Panels J–O would be easier to interpret if a legend were included directly in the figure.

Response to R4.12

A legend has been added directly within panels J–O of Figure 6, identifying reference (gray), NB mixture (blue), and simulation (red) traces.

R4.13 | Several figures: text too small

Several figures would benefit from larger text, especially plot titles. For example, the titles in Figure 6J–O are difficult to read at the current size.

Response to R4.13

We have increased font sizes for plot titles and axis labels throughout the figures to ensure readability at print size. Figures have been re-exported.

R4.14 | Non-convex objective: guidance on multiple initializations

Around line 761, the authors note that the objective is non-convex. Should users run CardamomOT with multiple initializations? Please provide guidance.

Response to R4.14

We have added practical guidance in Section 3 and in the online documentation:

1. For networks up to ∼20 genes with good temporal resolution, a single run is typically sufficient (Figure 4A–E shows convergence to similar GRN structures regardless of initialization).
2. For larger or sparser networks, we recommend 3–5 runs with different random seeds; high variability in edge weights across runs signals poor identifiability for that edge.

(see also our responses to: R1.2.)

We hope that the revised manuscript and this response letter satisfactorily address all concerns raised by the Editor and Reviewers. We remain available to provide any additional clarifications or analyses.

Sincerely,

Yann Maugé and Elias Ventre
