# Supplementary material for: CardamomOT: A mechanistic optimal transport-based framework for gene regulatory network inference, trajectory reconstruction and generative modeling
Source: PLoS Comput Biol. 2026 Oct 6;22(10):e1014838. doi: 10.1371/journal.pcbi.1014838 (PMC13686291; doi:10.1371/journal.pcbi.1014838)
Supplement: Peer review history

# Peer review: Decision Letter 0

**DOI:** 10.1371/journal.pcbi.1014838.r001

**Authors:** Marc Birtwistle, James Gallo

11 May 2026

CardamomOT: a mechanistic optimal transport-based framework for gene

regulatory network inference, trajectory reconstruction and generative modeling

Dear Dr. Ventre,

Thank you for submitting your manuscript to PLOS Computational Biology. After careful consideration, we feel that it has merit but does not fully meet PLOS Computational Biology's publication criteria as it currently stands. Therefore, we invite you to submit a revised version of the manuscript that addresses the points raised during the review process.

Please submit your revised manuscript by Jul 11 2026 11:59PM. If you will need more time than this to complete your revisions, please reply to this message or contact the journal office at ploscompbiol@plos.org. When you're ready to submit your revision, log on to https://www.editorialmanager.com/pcompbiol/ and select the 'Submissions Needing Revision' folder to locate your manuscript file.

Please include the following items when submitting your revised manuscript:

* A letter that responds to each point raised by the editor and reviewer(s). You should upload this letter as a separate file labeled 'Response to Reviewers'. This file does not need to include responses to formatting updates and technical items listed in the 'Journal Requirements' section below.

* A marked-up copy of your manuscript that highlights changes made to the original version. You should upload this as a separate file labeled 'Revised Manuscript with Track Changes'.

* An unmarked version of your revised paper without tracked changes. You should upload this as a separate file labeled 'Manuscript'.

Kind regards,

James Gallo

Academic Editor

PLOS Computational Biology

Marc Birtwistle

Section Editor

PLOS Computational Biology

**Journal Requirements:**

1) We ask that a manuscript source file is provided at Revision. Please upload your manuscript file as a .doc, .docx, .rtf or .tex. If you are providing a .tex file, please upload it under the item type u2018LaTeX Source Fileu2019 and leave your .pdf version as the item type u2018Manuscriptu2019.

2) Please upload all main figures as separate Figure files in .tif or .eps format. For more information about how to convert and format your figure files please see our guidelines:

https://journals.plos.org/ploscompbiol/s/figures

3) We notice that your supplementary Figures, and Tables are included in the manuscript file. Please remove them and upload them with the file type 'Supporting Information'. Please ensure that each Supporting Information file has a legend listed in the manuscript after the references list.

4) Please amend your detailed Financial Disclosure statement. This is published with the article. It must therefore be completed in full sentences and contain the exact wording you wish to be published.

- State the initials, alongside each funding source, of each author to receive each grant. For example: "This work was supported by the National Institutes of Health (####### to AM; ###### to CJ) and the National Science Foundation (###### to AM)."

- State what role the funders took in the study. If the funders had no role in your study, please state: "The funders had no role in study design, data collection and analysis, decision to publish, or preparation of the manuscript.".

If you did not receive any funding for this study, please simply state: u201cThe authors received no specific funding for this work.u201d

**Reviewers' comments:**

Reviewer's Responses to Questions

**Comments to the Authors:**

**Please note here if the review is uploaded as an attachment.**

Reviewer #1: SUMMARY

This manuscript presents CardamomOT, a mechanistic optimal-transport framework for inferring gene regulatory networks, reconstructing latent protein trajectories, and simulating temporal single-cell RNA-seq datasets. The method extends the authors’ previous CARDAMOM framework by incorporating exact time labels, protein kinetic priors, and an iterative inference scheme linking GRN calibration with trajectory reconstruction. The manuscript is technically ambitious and potentially valuable, but several claims would benefit from clearer qualification and additional analysis, particularly around benchmark fairness, uncertainty in inferred networks, and interpretation of perturbation predictions.

KEY FINDINGS AND SIGNIFICANCE

1. CardamomOT integrates mechanistic gene-expression modelling with optimal transport to jointly infer GRNs and reconstruct latent protein-level trajectories.

2. Synthetic benchmarks suggest improved GRN inference relative to several comparator methods, especially for directed and signed regulatory interactions.

3. The method can regenerate several features of experimental single-cell datasets, including broad expression structure, temporal cell-type proportions, and selected regulator dynamics.

4. The perturbation simulations suggest that the inferred models can generate biologically interpretable hypotheses, including predictions consistent with known reprogramming perturbations.

5. The work is significant because it moves trajectory inference beyond transcript-space transport alone and toward calibrated, mechanistically interpretable generative models.

MAJOR COMMENTS

1. Benchmark fairness should be clarified.

The synthetic data appear to be generated from a model closely related to the assumptions of CardamomOT. This is useful as a model-recovery test, but it may favour the proposed method. The authors should either add benchmarks with model misspecification or explicitly frame the synthetic benchmarking as validation under model-matched conditions.

2. Uncertainty in inferred GRNs should be reported.

Experimental GRNs are presented largely as point estimates. Because individual inferred edges are biologically interpreted, the authors should report edge stability or confidence, for example using bootstrapping, subsampling, repeated random initializations, or perturbation of kinetic priors.

3. Perturbation predictions should be more cautiously interpreted.

The distinction between predictions supported by independent experimental evidence and purely model-generated hypotheses should be made clearer. For the perturbation analyses, effect sizes and variability across repeated simulations would be more informative than reporting only highly significant chi-squared tests.

4. Claims moderation.

Several claims should be revised to distinguish validation on simulated data, where ground truth is available, from consistency checks on experimental data, where ground-truth GRNs and protein trajectories are not available. For example, the authors state that CardamomOT “accurately recovers velocity fields”, “accurately infers GRNs and reconstructs cellular trajectories”, and produces “reliable GRN structures”. These statements are appropriate for model-matched simulations but should be moderated when referring to experimental datasets. Please revise these statements so that simulation-based validation and experimental-data consistency are clearly separated.

MINOR COMMENTS

1. The authors use both “gene regulation network” and the more standard term “gene regulatory network”. Please choose one term and update the text for consistency, or explain what distinguishes the two terms and why they are needed.

2. The authors refer to their two-state gene-expression model as stochastic with respect to both M_i(t) and P_i(t). However, Eq. (2) and Figure 1A combine stochastic dynamics notation for M_i production with mean-field ODEs for M_i degradation and P_i dynamics. Could the authors please revise these equations using standard stochastic-dynamics notation. Otherwise, could the authors please explain this unique hybrid stochastic-deterministic notation.

3. Please define the notation \epsilon (\frac{k_{off}}{s_0}), e.g. does this denote an exponential distribution?

4. Please define the negative binomial parameterisation used in Eq. (3), since different conventions use different meanings for the second parameter.

5. I agree that the negative binomial distribution is appropriate here, as this is a well-established model for scRNA-seq count data. However, could the authors please provide an additional peer-reviewed source supporting this approximation, in addition to the PhD thesis currently cited [31]?

6. Apologies if my indexing is wrong, in Algorithm 1, should there be N-1 OT problems?

7. Please define abbreviations at first use, and then proceed with them consistently there after. E.g. the authors have written “Reference Fitting (RF). Reference Fitting”

8. Please state number of number of independent simulations and what error bars/bounds represent in figures captions.

9. For Figure 7, please provide more detail on how reconstructed cell counts were obtained for the chi-squared tests. E.g. are all p< 10^-3 because you ran 1000 simulations?

10. I am dubious that the term “digital twin” is appropriate here, as the model does not appear to involve iterative updating between the model and the biological system. Although the authors provide a definition, this does not seem to align with standard definitions of digital twinning. Could the authors please moderate this language?

11. Please ensure that all experimental datasets have clear accession numbers to the original data. This should be included in Data and Code Availability section in addition to the GitHub link. I did not see this in the git hub README or data set folders. Perhaps I missed it.

Reviewer #2: Comment 1: Figure 1 could be cleaned up: it appears to be a jarring mix of vector text and raster equations of varying resolution, similarly B seems to have vector axes but low-resolution raster points.

Comment 2: I am unsure what the Figure 1 plots in B and E are meant to represent in relation to each other. I would have thought these two plots would be switched to highlight the extended predictive capabilities of the generative model, but E (predictive trajectories) seems to be a subset of the data points of B (experimental plot).

Comment 3: In Figure 3, the ‘Trees’ box border has been clipped.

Comment 4: A considerable emphasis is placed on the model’s extension to a non-linear regime. Is there a flaw in the methodology from discretising the trajectories into linear segments using the piecewise constant parameter alpha as described in Appendix A? It seems that the justification for this lies in the asymptotic regime where the timescale of protein dynamics << mRNA degradation, but what about outside this regime?

Comment 5: Similarly, the linear-spaced sample set (line 904) raises a couple of questions due to the userparameter dependency: a) how is n_pas determined in practice b) to what effect does a coarse sample space (in particular, supposing that the smallest alpha is the smallest element of the sample set) have on the misidentification of candidate cells, and therefore the trajectory?

Comment 6: The choice of the timestep influences the switching parameter sample set and therefore determined alpha. Section 2.1 describes the timestep as a user-parameter, and importantly it is used in generating appropriate datasets via Harissa. This suggests to me that bias could be introduced in the datasets used for the method comparison as appropriate temporal snapshots and alpha (n_pas) could be pre-tuned to suit the dataset. a) I would like there to either be more detail on the choices of both times and n_pas (switching parameter sample set) used in the benchmark dataset generation. b) I would like to see an analysis of the sensitivity of the results presented in Figure 3 under timestep and n_pas refinement, covering both coarse and (computationally feasible) fine parameter values.

Comment 7: Following the discussion of results in Figure 3 of Section 2.3, should the reader conclude that CardamomOT currently implements the best method for GRN inference? Therefore it follows that Cardamom is the second best method? I believe this section requires a more nuanced discussion. As a reader, I find it difficult to discern what is responsible for this hierarchy. I am left reading that the conclusion I should make is that optimal transport minimising the (squared-)Euclidean cost is worse than neglecting the optimal transport mechanics altogether.

Reviewer #3: This manuscript introduces CardamomOT, a framework that combines mechanistic modeling with optimal transport to jointly infer gene regulatory networks, cellular trajectories, and a generative model from time-series scRNA-seq data. The main claims are well motivated and, in my view, of clear relevance to the field. The integration of these components is novel relative to prior work, including the authors’ earlier CARDAMOM method, and represents a meaningful methodological advance.

The approach is carefully constructed and generally rigorous, and the results on simulated benchmarks are convincing. The application to experimental datasets is promising, although the support there is somewhat more qualitative. It would strengthen the manuscript to include more quantitative evaluation on real data where feasible, as well as a brief discussion of sensitivity to key hyperparameters and model assumptions.

The manuscript is well organized and the methods are described in sufficient detail to allow reproduction, with code and data appropriately shared. Overall, I find this to be a strong contribution. I recommend acceptance after minor revision.

Reviewer #4: # “CardamomOT: a mechanistic optimal transport-based framework for gene regulatory network inference, trajectory reconstruction and generative modeling”

The authors present CardamomOT, a mechanistic optimal transport-based framework for

inferring gene regulatory networks from time-series scRNA-seq data. Once calibrated, the

inferred model can also be used for trajectory reconstruction, generative modelling, and in

silico perturbation analysis. Inferring gene regulatory networks from temporal single-cell

data is an increasingly more important problem, and this work is very timely, with clear

applications in areas such as developmental biology.

I believe the manuscript would benefit from additional validation, clearer discussion of

some assumptions, and improved presentation of several methodological details. I have

separated my comments into major and minor points.

## Major comments

### 1. Robustness of low-dimensional embedding validation

Several validation analyses rely on visual comparison in UMAP space between reference and

simulated data. While UMAP is widely used and useful for visualization, it is known that the

apparent preservation of local and global structure can depend on parameter choices and on

the embedding method. This is particularly relevant here because the datasets have an

inherent temporal or trajectory-like structure.

I therefore suggest that the authors repeat the key low-dimensional visualization analyses

using an additional better trajectory-preserving dimensionality reduction method, such as

PHATE (https://www.nature.com/articles/s41587-019-0336-3). This could be included as a

supplementary figure if space is limited.

### 2. Gene subset selection and possible selection bias

For the experimental datasets, the analysis is performed on selected subsets of genes,

either from previous studies or based on marker/dynamic-gene criteria. Because GRN inference

can be sensitive to the choice of included genes (and prior biases can be introduced through

sub-setting genes), the manuscript would benefit from a clearer discussion of how this

subsetting may affect the inferred networks.

It would also be useful to know whether CardamomOT can be run on substantially larger gene

sets, and whether the main limitation is computational runtime (as suggested by the current

runtimes), identifiability, or both.

### 3. Accuracy and availability of kinetic parameters in realistic applications

A central part of CardamomOT is its incorporation of kinetic parameters, especially protein

degradation rates. However, the method requires prior knowledge of these rates, and the

performance depends to some extent on their accuracy. The robustness analysis in Figure 4F

is helpful, but it would be useful to connect this analysis more explicitly to realistic

biological settings.

For example, what level of uncertainty is typical for protein degradation-rate estimates in

the systems considered here? Do current experimental or literature-derived estimates

generally fall within the uncertainty range where the method remains robust?

### 4. Additional perturbation predictions for less-characterized genes

The perturbation analyses are one of the most interesting aspects of the manuscript,

especially because the model can generate mechanistically interpretable predictions. The

examples involving Dnmt3a, CHGA/STMN2, Obox6, and Zfp42 are really good illustrations!

To further demonstrate the discovery potential of the framework, could the authors provide

at least a proof-of-concept showing that CardamomOT can identify less-characterized genes

with potentially important regulatory roles? Experimental validation of such predictions

would naturally be beyond the scope of the present work, but providing a ranked list or

selected examples of candidate genes would help illustrate how CardamomOT can be used to

generate novel, testable biological hypotheses.

### 5. Clarification of simulated-data noise model

The simulated benchmarks are valuable because they allow comparison with ground-truth

networks and protein trajectories. However, it is not fully clear whether the simulations

include technical noise resembling scRNA-seq measurement noise, beyond the intrinsic

stochasticity of the mechanistic gene-expression model.

Could the authors clarify whether the simulated datasets include measurement noise? If not,

I suggest adding a robustness analysis in which technical noise is introduced into the

simulated data. This would strengthen the benchmark and help establish how robust the method

is under more realistic single-cell sequencing conditions.

### 6. Usability and documentation of the software package

It is great that the authors provide code and data through a GitHub repository. However, the

documentation is currently somewhat difficult to navigate, as many documentation files

appear to be separate markdown files in the root repository.

I suggest that the authors consider hosting structured online documentation, for example

using ReadTheDocs. A clearer separation between installation instructions, tutorials, API

documentation, and worked examples would make the method easier to use and reproduce.

## Minor comments

1. Around line 168, the text describes the stochastic interplay between gene activation,

mRNA synthesis, and protein production. Since mRNA and protein degradation are also

central components of the model, should it also be mentioned here?

2. Around line 180, the manuscript assumes the bursty regime. Is this expected to hold for

all genes included in the model? If not, what fraction of genes can reasonably violate

this assumption before performance is affected?

3. Around line 302, the Neural ODE recalibration step is difficult to follow. It would be

easier to follow the manuscript if more details on the Neural ODE part could be provided.

4. Around line 460, the statement that sparse networks with hub regulators are a hallmark of

biologically realistic network structure should be supported by a reference.

5. The section beginning around line 453 is quite long and contains several important

results on experimental GRN inference without a main-text figure. The authors could

consider shortening this section or moving one of the relevant supplementary figures into

the main text to improve readability.

6. In Figure 6, panels J–O would be easier to interpret if a legend were included directly

in the figure.

7. Several figures would benefit from larger text, especially plot titles. For example, the

titles in Figure 6J–O are difficult to read at the current size.

8. Around line 761, the authors note that the objective is non-convex and that convergence

to a global optimum is not guaranteed. Given this, should users run CardamomOT with

multiple initializations in practice? Please provide guidance on whether multistart runs

are recommended, and whether variability across runs should be used as a diagnostic for

identifiability.

**********

**Have the authors made all data and (if applicable) computational code underlying the findings in their manuscript fully available?**

The PLOS Data policy requires authors to make all data and code underlying the findings described in their manuscript fully available without restriction, with rare exception (please refer to the Data Availability Statement in the manuscript PDF file). The data and code should be provided as part of the manuscript or its supporting information, or deposited to a public repository. For example, in addition to summary statistics, the data points behind means, medians and variance measures should be available. If there are restrictions on publicly sharing data or code —e.g. participant privacy or use of data from a third party—those must be specified.

Reviewer #1: Yes

Reviewer #2: Yes

Reviewer #3: Yes

Reviewer #4: Yes

**********

PLOS authors have the option to publish the peer review history of their article (what does this mean?). If published, this will include your full peer review and any attached files.

If you choose “no”, your identity will remain anonymous but your review may still be made public.

**Do you want your identity to be public for this peer review?**  For information about this choice, including consent withdrawal, please see our Privacy Policy.

Reviewer #1: No

Reviewer #2: No

Reviewer #3: **Yes:**  Yudou He

Reviewer #4: No

[NOTE: If reviewer comments were submitted as an attachment file, they will be attached to this email and accessible via the submission site. Please log into your account, locate the manuscript record, and check for the action link "View Attachments". If this link does not appear, there are no attachment files.]

**Figure resubmission:**

**Reproducibility:**

?>

# Author comment: Author response to Decision Letter 1

**DOI:** 10.1371/journal.pcbi.1014838.r002

31 Aug 2026

Attachment Submitted filename: response_letter.pdf

# Peer review: Decision Letter 1

**DOI:** 10.1371/journal.pcbi.1014838.r003

**Authors:** Marc Birtwistle, James Gallo

21 Sep 2026

Dear Dr. Ventre,

We are pleased to inform you that your manuscript 'CardamomOT: a mechanistic optimal transport-based framework for gene

regulatory network inference, trajectory reconstruction and generative modeling' has been provisionally accepted for publication in PLOS Computational Biology.

Before your manuscript can be formally accepted you will need to complete some formatting changes, which you will receive in a follow up email. A member of our team will be in touch with a set of requests.

Please note that your manuscript will not be scheduled for publication until you have made the required changes, so a swift response is appreciated.

IMPORTANT: The editorial review process is now complete. PLOS will only permit corrections to spelling, formatting or significant scientific errors from this point onwards. Requests for major changes, or any which affect the scientific understanding of your work, will cause delays to the publication date of your manuscript.

Should you, your institution's press office or the journal office choose to press release your paper, you will automatically be opted out of early publication. We ask that you notify us now if you or your institution is planning to press release the article. All press must be co-ordinated with PLOS.

Thank you again for supporting Open Access publishing; we are looking forward to publishing your work in PLOS Computational Biology.

Best regards,

James Gallo

Academic Editor

PLOS Computational Biology

Marc Birtwistle

Section Editor

PLOS Computational Biology

***********************************************************

Reviewer's Responses to Questions

**Comments to the Authors:**

**Please note here if the review is uploaded as an attachment.**

Reviewer #1: The authors have sufficiently addressed all of the comments I raised, and I now believe that this manuscript is suitable for publication.

Reviewer #2: The work represents a valuable model and accompanying tool. The authors have responded well to my comments and clarified key aspects of the work to the reader, particularly regarding the assumptions and limitations in their derivation.

In this manuscript, the authors present a model and accompanying software, CardamomOT, designed to advance the field of gene regulatory network inference in time-referenced datasets. They do so by considering the optimal transport of gene expression outside of the current, non-physical linear (Brownian) regime into a non-linear regime. An interesting feature of this work is the ability of the final, output model to behave in a generative manner and create new (derivative) datasets of existing experimental datasets.

The review of existing literature and the model derivation are explained well. Alongside the CardamomOT software, these support the work as a valuable addition to the field.

My major concerns with the original submission were visual presentation of figures, reliability of results and ambiguity of model assumptions and limitations. In the revised submission, I believe these have been addressed, with the model now presented with greater clarification and detail to the reader.

Therefore, after reviewing the revised manuscript, it is my view that the authors have satisfactorily responded to my comments and the manuscript has been substantially improved. I have no further comments.

Reviewer #4: I thank the authors for thoroughly addressing all my comments, especially setting up a good documentation and for extensively discussing tunable parameters such as gene-set selection.

**********

**Have the authors made all data and (if applicable) computational code underlying the findings in their manuscript fully available?**

The PLOS Data policy requires authors to make all data and code underlying the findings described in their manuscript fully available without restriction, with rare exception (please refer to the Data Availability Statement in the manuscript PDF file). The data and code should be provided as part of the manuscript or its supporting information, or deposited to a public repository. For example, in addition to summary statistics, the data points behind means, medians and variance measures should be available. If there are restrictions on publicly sharing data or code —e.g. participant privacy or use of data from a third party—those must be specified.

Reviewer #1: Yes

Reviewer #2: Yes

Reviewer #4: Yes

**********

PLOS authors have the option to publish the peer review history of their article (what does this mean?). If published, this will include your full peer review and any attached files.

If you choose “no”, your identity will remain anonymous but your review may still be made public.

**Do you want your identity to be public for this peer review?**  For information about this choice, including consent withdrawal, please see our Privacy Policy.

Reviewer #1: No

Reviewer #2: No

Reviewer #4: No

# Editor report: Acceptance letter

**DOI:** 10.1371/journal.pcbi.1014838.r004

**Authors:** Marc Birtwistle, James Gallo

PCOMPBIOL-D-26-00826R1

CardamomOT: a mechanistic optimal transport-based framework for gene

regulatory network inference, trajectory reconstruction and generative modeling

Dear Dr Ventre,

I am pleased to inform you that your manuscript has been formally accepted for publication in PLOS Computational Biology. Your manuscript is now with our production department and you will be notified of the publication date in due course.

The corresponding author will soon be receiving a typeset proof for review, to ensure errors have not been introduced during production. Please review the PDF proof of your manuscript carefully, as this is the last chance to correct any errors. Please note that major changes, or those which affect the scientific understanding of the work, will likely cause delays to the publication date of your manuscript.

Soon after your final files are uploaded, unless you have opted out, the early version of your manuscript will be published online. The date of the early version will be your article's publication date. The final article will be published to the same URL, and all versions of the paper will be accessible to readers.

For Research, Software, and Methods articles, you will receive an invoice from PLOS for your publication fee after your manuscript has reached the completed accept phase. If you receive an email requesting payment before acceptance or for any other service, this may be a phishing scheme. Learn how to identify phishing emails and protect your accounts at https://explore.plos.org/phishing.

Thank you again for supporting PLOS Computational Biology and open-access publishing. We are looking forward to publishing your work!

With kind regards,

Sharmila Kamatchi

PLOS Computational Biology | Carlyle House, Carlyle Road, Cambridge CB4 3DN | United Kingdom ploscompbiol@plos.org | Phone +44 (0) 1223-442824 | ploscompbiol.org | @PLOSCompBiol
